# Fundamental challenges in assessing the impact of research infrastructure

**DOI:** 10.1186/s12961-021-00769-z

**Published:** 2021-08-18

**Authors:** Sana Zakaria, Jonathan Grant, Jane Luff

**Affiliations:** 1 Central Commissioning Facility, National Institute of Health Research, 15 Church Street, TW1 3NL Twickenham, United Kingdom; 2 Policy Institute, King’s College London, SE1 8WA London, United Kingdom

**Keywords:** Impact assessment, Research infrastructure, Evaluation, Realist evaluation, Pipeline, Platform, COVID-19, Impact frameworks, Time lags, NIHR BRC

## Abstract

Clinical research infrastructure is one of the unsung heroes of the scientific response to the current COVID-19 pandemic. The extensive, long-term funding into research support structures, skilled people, and technology allowed the United Kingdom research response to move off the starting blocks at pace by utilizing pre-existing platforms. The increasing focus from funders on evaluating the outcomes and impact of research infrastructure investment requires both a reframing and progression of the current models in order to address the contribution of the underlying support infrastructure. The majority of current evaluation/outcome models focus on a “pipeline” approach using a methodology which follows the traditional research funding route with the addition of quantitative metrics. These models fail to embrace the complexity caused by the interplay of previous investment, the coalescing of project outputs from different funders, the underlying infrastructure investment, and the parallel development across different parts of the system. Research infrastructure is the underpinning foundation of a project-driven research system and requires long-term, sustained funding and capital investment to maintain scientific and technological expertise. Therefore, the short-term focus on quantitative metrics that are easy to collect and interpret and that can be assessed in a roughly 5-year funding cycle needs to be addressed. The significant level of investment in research infrastructure necessitates investment to develop bespoke methodologies that develop fit-for-purpose, longer-term/continual approach(es) to evaluation. Real-world research should reflect real-world evaluation and allow for the accrual of a narrative of value indicators that build a picture of the contribution of infrastructure to research outcomes. The linear approach is not fit for purpose, the research endeavour is a complex, twisted road, and the evaluation approach needs to embrace this complexity through the development of realist approaches and the rapidly evolving data ecosystem. This paper sets out methodological challenges and considers the need to develop bespoke methodological approaches to allow a richer assessment of impact, contribution, attribution, and evaluation of research infrastructure. This paper is the beginning of a conversation that invites the community to “take up the mantle” and tackle the complexity of real-world research translation and evaluation.

**Supplementary Information:**

The online version contains supplementary material available at 10.1186/s12961-021-00769-z.

## Introduction

The scientific response to the COVID-19 pandemic has been unstinting. Within a matter of days, researchers around the world—in public and private settings—mobilized to sequence SARS-CoV-2 (the virus that causes COVID-19) [1], began the development of vaccines [2], tested the use of various steroids to improve outcomes [3], and developed citizen science networks for population surveillance [4]. At the time of writing, some 15 months after the virus emerged in China [5], a variety of vaccines using different technologies are being used to protect populations from the acute, and often fatal, respiratory disease COVID-19. This is an extraordinarily fast scientific development, given that historically it takes about 17 years for research to translate from bench to bedside. The reason for this, as discussed by Hanney et al. (2020), is that given the serious public health emergency, the classic “pipeline” or programmatic model of linear innovation was abandoned, in favour of a faster but most likely more expensive approach of parallel working where various activities were undertaken at the same time, including the manufacture of vaccines before their safety and efficacy were proven [6]. These multiple strands of activities were supported by underlying research infrastructures (RI)—or “platforms”, as we refer to them in this paper. As explored in this paper, one of the main reasons that vaccine development was so quick was because, critically, a number of underlying RIs/platforms existed before the emergence of SARS-CoV-2.

**Fig. 1:**
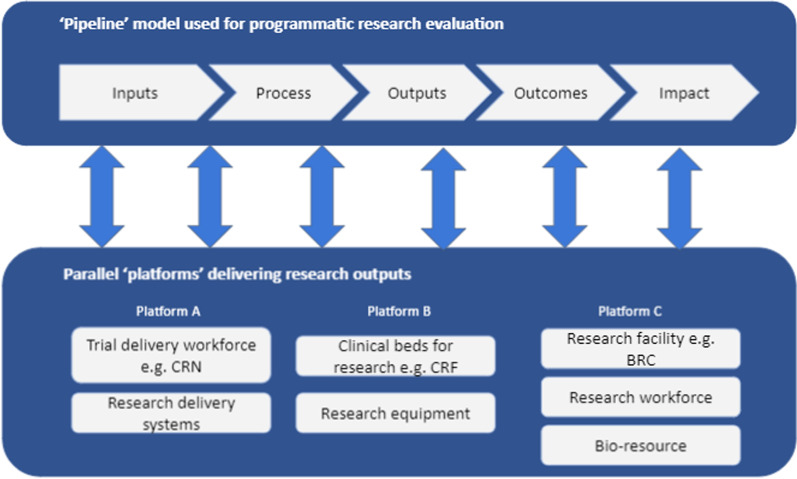
Schematic diagram illustrating the difference between a pipeline model of evaluation and the platform models of research production

Given the economic hardships that will ensue in the post-pandemic environment, governments are likely to be under pressure to review and scrutinize research allocation. Roope and colleagues shed light on the importance of RI investment and caution against short-termism in resource allocation whilst making a case for developing a framework that allows the value of this investment to be surfaced in the public eye [7]. The complex contributions played by different parts of the research system may be better described by progressing the current research evaluation model from one characterized by a “pipeline” to one better described as a “platform” (as illustrated in Fig. 1).

It should be noted that the purpose of Fig. 1 is neither to present a conceptual nor an empirical depiction of the difference between the two approaches, but to schematically illustrate some of the differing characteristics. The top panel in Fig. 1 illustrates a classic logic model, whereby the research impact would be evaluated through a series of “if … then” statements. For example, if a funder supported a project (inputs), then a hypothesis could be investigated. If this hypothesis was proven (process), then it would be written up in a research paper (output). If that output was, for example, cited in a clinical guideline (outcome), then it could lead to longer and healthier lives (impact).

This theory underpins the majority of research evaluations [8], but as illustrated in the bottom half of Fig. 1, is not suited to holistically account for investments into underlying platforms and their complex interactions—whether they are bio-resource, skilled people, research equipment, or collaboration with complementary infrastructure (networks). Multiple platforms may work in parallel, working across the research ecosystem and utilizing the collective outputs of multiple research projects as indicated by the bidirectional arrows in Fig. 1. Although one may argue that investment into infrastructure could be captured as “inputs” and so on, this model is reductionist in its ability to account for the complex interactions between multiple infrastructures and taking into account the existing knowledge that has been produced/available for use. Simply stating investment into RI as inputs limits a holistic view of the added value of RI. For instance, platforms A, B, and C, as denoted in Fig. 1, all worked in parallel and in collaboration to deliver the Oxford University/AstraZeneca vaccine—AZD1222; the vaccine relies on the delivery of genetic material via a viral vector which acts as a carrier to stimulate an immune response. In the case of AZD1222, the vector carries code for the SARS-CoV-2 spike protein [9]. The Pfizer and Moderna vaccines also focus on the spike protein, but use alternative technology based on messenger RNA (mRNA) to trigger an immune response. However, both approaches are based on decades of research, and critically having in place existing “platforms” meant that candidate vectors and mRNA (both bio-resources) could quickly be adapted for the specific genetic profile of SARS-CoV-2 [10, 11]. When it comes to evaluating the impact of these advances, it would be inappropriate to focus solely on the programmatic pipeline—that is, the development of the vaccine since January 2020, or the investment into the current cycle of RI—without including the pre-existing platforms or the parallel contributions from different RIs (Biomedical Research Centres [BRCs], Clinical Research Facilities [CRFs], Clinical Research Network [CRN]) that enabled such rapid scientific progress.

A notable example of a platform model is one exemplified by National Institute for Health Research (NIHR)-funded BRCs which link closely with clinical trial delivery partners (NIHR CRFs). In England, these clinical RI centres are contracted with National Health Service (NHS) Trusts primarily to fund the underlying support mechanisms that are required to deliver clinical-, health-, and care-related research. The NIHR infrastructure funding provides long-term support towards the cost of delivering early-phase experimental medicine research across England. This includes support towards the salaries of skilled research professionals, collaborations, and funding for services and facilities [12]. First awarded in 2007, these platforms have provided targeted and strategic investment to support world-leading research in health and care and have been crucial in pioneering first-in-human studies and progressing novel treatments for patient benefit.

Again, when assessing the swiftness and magnitude of the response to the COVID-19 pandemic, the role played by NIHR BRCs and CRFs, as part of the multiple enabling platforms, has been crucial. For example, the Randomised Evaluation of COVID-19 Therapy (RECOVERY) Trial, which has been identifying treatments for people hospitalized with COVID-19, proceeded at a rapid speed with patients enrolled 9 days after the study protocol was drafted. This was a national endeavour, coordinated largely through the NIHR CRN, involving 176 acute hospital trusts including all of the NIHR BRCs. Most notably, the researchers supported by NIHR Oxford BRC found that dexamethasone, an inexpensive and widely available drug, cut the risk of death by a third for patients on ventilators, and by a fifth for those on oxygen [3].

NIHR BRCs played an equally monumental role in developing a vaccine for COVID-19. When the virus emerged at the end of 2019, the BRC Vaccines research theme team at Oxford was already working on human coronavirus vaccines and was in a unique position to rapidly respond to the pandemic. The vaccine candidate progressed rapidly to phase III clinical trials across 19 trial sites in the United Kingdom, South Africa, and Brazil within the space of weeks [2].

These examples highlight the role of established platforms in being able to leverage expertise, facilities, multidisciplinary teams with dedicated personnel, and pre-existing strategic partnerships with industry (in this case AstraZeneca) to deliver at pace, on a global scale. It is highly unlikely that this would have been the case had the infrastructure not been in place. This counterfactual argument would be hard to establish using traditional approaches to assessing research impact and evaluating research outputs and outcomes. It is our impression that research impact assessment often excludes underlying infrastructures and platform contributions, and that this is confirmed to a degree by our selective scan of the literature, but it would be important to empirically try to test that assumption in due course.

The purpose of this paper is to explore the challenges of assessing the impact of RI—or platforms as we refer to them in this paper—and to define a methodological agenda to improve such evaluations in the future. To do this, we provide a brief and selective review of the literature (methodology for review provided as a Additional files 1 and 2) on the limited approaches for assessing the impact of RI, and from that review and our experience in the field, most notably an internally led review of NIHR-funded BRCs and CRFs, identify key challenges that need to be addressed. We conclude with some reflections on what this means for the field of research impact assessment.

## How are RIs traditionally addressed?

Research impact assessment is “a growing field of practice that is interested in science and innovation, research ecosystems and the effective management and administration of research funding” [13]. The practice of evaluating the impact of RIs has been gathering momentum and evolving over the last decade. With increasing demand from stakeholders (e.g. funders, government treasuries, and the public/taxpayers) to understand the value of RI, there has been an increasing focus on quantifying and qualifying the impact of investing in these platforms. For the purpose of this paper, we are borrowing the European Strategy Forum on Research Infrastructure (ESFRI) definition of RI:

facilities, resources and related services that are used by the scientific community to conduct top-level research in their respective fields and covers major scientific equipment; knowledge-based resources such as collections, archives or structures for scientific information; enabling Information and Communications Technology-based infrastructures such as Grid, computing, software and communication, or any other entity of a unique nature essential to achieve excellence in research. Such infrastructures may be ‘single-sited’ or ‘distributed’. [14]

Although this is a broad definition of RI, it encapsulates most of the relevant aspects of a clinical RI funded through NIHR such as BRCs, CRFs, and the CRN. The NIHR makes a significant investment in clinical infrastructure each year. The 2018/19 annual report indicates that £622 m (more than 50% of the annual budget) was used to support clinical RI.

Much of the select literature analysed made insightful observations about large-scale, technology-driven global infrastructure, such as those encompassed by the ESFRI programme, its distinct phases, and the varied evaluation needs associated with each phase [15–17]. In addition, there has been much discussion of how the context and type of RI affects impact assessments—for instance, whether RI is virtual or a single site, or for basic science or applied research [18].

There have been accounts of use of multiple evaluation models and assessment frameworks, ranging from Dilts’ “three-plus-one” evaluation model to WHO’s eight evaluative principles, Dozier's use of social network analysis, Davies and Dart’s most significant change theory, the Payback Framework, and Donovan and Hanney’s social impact assessment methods [19–23]; however, most of these models of assessment are built to suit a programmatic pipeline model of progression of research. We are taking a simplistic view of linear models of assessment for effect; work from Hanney and colleagues used the Payback Framework to assess the value of RI by reviewing networks and the absorptive capacity of the research system; however, this is still the least-studied aspect of the framework. Moreover, in practice, the application of frameworks is led by pragmatism [8] which can mean these important nuances can often be overlooked when using programmatic frameworks for assessing RI. In fact, there is much literature discussing the limitations of utilizing logic model-based frameworks in accounting for complexity and interactions, and there is a recognition that the traditional logic model needs to evolve into something more dynamic [24].

The use of cost–benefit analysis (CBA) and cost-effectiveness analysis (CEA) methodologies to quantify benefits of infrastructure in particular has been the most favourable approach and remains so to this day, especially when articulating benefits to the Government and the Treasury [25–27], despite the challenges around monetizing the value of health and the quality of life. The clinical RI in the United States, the Clinical and Translational Science Awards (CTSA) Consortium, have conducted a series of evaluations to articulate the benefits of RI and have managed their portfolio by defining consistent terminology of inputs, outputs, and outcomes to collect data that can be harmonized and compared across the national portfolio of 62 centres [25]. In recent years, a large amount of evaluative techniques have focussed on bibliometric network analysis and a structured use of case studies/qualitative analyses, exemplified in the Research Excellence Framework (REF) exercise and the ACCELERATE framework [26, 27]. A recently emerging modular approach provided by the RI paths project also provides an interesting lens whereby the modular approach allows the user flexibility in tailoring the evaluative approach to select aspects that are important to focus on [28]. In doing so, it addresses some of the challenges being raised in that it starts breaking out of the mould of a traditional pipeline model of evaluation.

However, as mentioned earlier, most of the literature we reviewed was geared towards assessing the impact of RI in the context of a “pipeline” model which is borrowed and adapted from assessing research grants and programmes rather than RI per se. The evaluation models and metrics blur the pipeline and platform models and do not draw a clear enough distinction. The nuances and complexity of a “platform” model, where utilization of expertise and facilities, delivery of team science, and fostering of innovation translates into benefits for the population, is not typically addressed through any of the tools and methods mentioned in the literature. Although methods like CBA or the modular approach provided by the RI paths project are needed, they need to be complemented by metrics and techniques that can surface the value of RI in the context of a platform model to articulate benefits such as the development of a COVID-19 vaccine within 12 months, facilitated by support from NIHR BRCs, CRFs, CRN, and commercial partners, among others.

Additionally, one of the biggest challenges identified in the literature is around assessing impact of RI with respect to time lags and the challenge of contribution/attribution. None of the methods can account for this; rather, the literature calls for charting realistic milestones over the course of the life cycle of a RI, instead of tracking every infrastructure-supported project over a course of a typical 17 years—a rough projection of the time taken to translate advances from bench to bedside.

## Challenge 1: Traditional criteria for assessing the impact of RI are not fit for purpose

Despite the multitude of frameworks and methodologies devised to support funders and recipients of funds to evidence the value of RI, it remains a subjective and challenging task, as no single methodology or concept serves all stakeholders’ needs nor does it reconcile the platform and pipeline model dichotomy. The challenge is further compounded when resource allocation, in a fiercely competitive environment, is primarily based on an allocation system that values project-based funding approaches. We are going to frame this challenge through the lens of contribution/attribution, time lags, and marginality or nuanced differences.

From the viewpoint of the contribution/attribution challenge, some postulate that public investment in RIs is justified given the multifaceted role they play in advancing our knowledge, innovating, driving inward investment/economic growth, and building capacity [29], whilst others have taken a view that there is no standardized evidence to attest to these claims [30]. Despite the undeniably crucial role played by RI in tackling the COVID-19 pandemic, most criteria of assessing infrastructure are not suited to disentangling the contribution of RI in outcomes and impact achieved. Most outputs and outcomes claimed by infrastructure are also claimed by project grants with little or no assessment of the unique elements that have been supported by RI. Contribution analysis methodology (and quantification of contribution) is well established and can be deployed here; however, it is based on the premise of a linear pipeline model and thus is perceived as such and only utilized in that manner [31]. There is a need to establish contribution analysis suited to RI so that the unique aspects and benefits of infrastructure can be articulated.

Although many theories and indicators are emerging, uniquely placed for assessing RI [28, 32, 33], academically accepted impact assessment metrics are primarily the yardstick with which the success of RI is measured. These “prestige” metrics such as citation counts are often culturally accepted by funding organizations with a substantive focus on “volume” and metrics that are easy to collect and count. Focusing on the number of patients recruited or numbers of trainees trained means that quite often the value of undertaking such activities is neither questioned nor surfaced. RIs can be compared against each other on the basis of these criteria which do not address the fundamental differences in the strategic purpose each play in the translational research landscape. A large BRC may produce more papers than a smaller BRC for instance; however, it tells us little about the significance of these contributions in their respective fields. BRCs are established to drive research into the clinic; CRFs are delivery vehicles for the progression of early-phase or first-in-class trials.

More focus is needed on assessing the value of innovation, team science, and encouraging the research community to review RI through the lens of complex and system-level change, linking up as clusters to propagate regional and national health research agendas. Evaluations commissioned for RI should look beyond economic returns/regional multipliers (as important as they are) and the traditional “pipeline metrics” to attest to the value of RI as a platform. The REF and the Knowledge Exchange Framework (KEF), which look at benefits beyond academia, the emergence of the responsible metrics movement, and reducing waste in research agendas, all provide a meaningful lens through which RI impact assessments criteria can be focussed and improved. The use of qualitative analyses can support the reframing of RI in the context of a platform, recognizing the added value it provides in fostering innovation and high-risk research.

Lastly, given the time lags of translating research into patient benefit, quite often there is a disconnect between the criteria of assessment and the time frame within which it is warranted. There needs to be a reframing of the kind of outputs and outcomes that should be assessed in relation to RIs and their life cycle.

## Challenge 2: Despite the long-term nature of infrastructural investments, research impact assessments are often undertaken in unrealistic timescales

One of the most talked about aspects of impact assessments, especially in biomedical research, are time lags. Time lags are widely debated in terms of agreeing upon models of assessment and what constitutes the starting point for a particular intervention/innovation [34, 35].

Time lags are of particular interest when assessing RI due to the premise that investing in platforms like NIHR-funded BRCs will expedite the translation of biomedical research (i.e. translate lab-based science into human application) and bridge the T1 gap (whereby T stands for translation, and 1 denotes the first phase of translational research) [36]. Understanding what affects time lags is complex and multifaceted which is why a systematic approach is rarely applied across a health system to understand changes in translation timelines. Multiple studies have found that factors like political pressure, research community engagement, and funder clout are all contributing factors to expediting time lags in research translation [37]. COVID-19 vaccine development, for instance, provides a classic example of an expedited translation event due to political pressure and increased access to rapid funding due to the acute nature of the problem being addressed.

Let us use COVID-19 vaccine development as an example to highlight the complexity of time lags and to reflect on appropriate timescales for impact assessments of RI. Although the delivery of the vaccine itself has been rapid compared to vaccine development in other areas, the technology of utilizing viral vectors and mRNA spike proteins had already been established some years ago. The two key challenges emerging here are determining what constitutes the start point of this particular intervention and at what time points can appraisals of outcomes and impact take place? When vector technology was developed, the assessment of its impact could not have truly taken place as the technology continues to be utilized and the magnitude of its impact has been increasing over time.

Hence, one of the biggest challenges of impact assessments in RI is evidencing expected outcomes and impact within one funding cycle of investment (typically 5 years in the NIHR). There is a need to determine what the expected outcomes and impact should be for the duration of an award cycle and what impact can be expected over a longer time period of continued investment.

It is therefore important to ensure that in the short-term the evidence collected for the purposes of understanding impact are made up of value indicators as discussed in Challenge 1 rather than solely focussing on metrics that accrue quickly and are easy to capture. It may even warrant development of hypothetical scenarios and “projected impact”, which is currently not the desired choice of evidence by United Kingdom funders and government bodies.

In addition, creating a shared expectation of long-term outcomes and impact and defining timelines for that can enable systematic evaluations to take place every 10–15 years against those expectations with the caveat that long-term impacts may continue to accrue outside of this assessment period. This, however, requires acceptance of the need for a longer-term view to allow benefit to accrue and a distinction between what is meaningful to measure rather than what is obtainable. It also requires planning for undertaking impact assessments over a longer time frame than an annual setting.

## Challenge 3: There is limited appetite and opportunity for innovating new and appropriate criteria and methods for assessing the impact of RI

One of the interesting reflections in reviewing the select literature on research evaluation is how it is so strongly embedded in the theoretical framework of logic modelling. To a degree, this is understandable as the research *funding* process is itself a series of linear steps that naturally follow the logic model of inputs, process, outputs, outcomes, and impact. However, it is also the case that the innovation literature is quite clear that the *research* process is itself not linear [38], and as discussed above, this is especially the case for the contribution of research platforms.

Broadly speaking, there are three dominant evaluation paradigms: logic models; systems-based approaches; and realist evaluation [39]. Systems-based approaches allow for a complex and dynamic set of interactions to assess how “a set of things work together as a whole” [40]. Given their inherent messiness, systems evaluation approaches combine multiple methods and data sources to build up a view of the “whole” and the contribution that different components make to that whole, including in this case, research platforms. Realist evaluation adopts a “context–mechanism–outcome” (CMO) framework and is based on understanding what works in what contexts, how, and for whom (i.e. in “real” life) rather than does it work [41]. In essence, such evaluations focus on understanding how different mechanisms of an intervention result in change and, critically, what contextual factors will influence that mechanism in determining outcomes, and variations in those outcomes. As such, the realist approach or the systems approach may be more appropriate for assessing the impact of RI and capturing the nuances and complex interactions of the platform model, as illustrated in Fig. 1, than the more traditional logic model approach. One of the advantages of the realist and systems approaches (over logic models) is that there is more focus on relationships and power which, in the context of COVID-19, may prove to be an important enabler. For example, pre-existing relationships between the scientific community, science and medical advisors, and the political and decision-making elite seem critical in the rapid start-up of the RECOVERY trials.

It is interesting that in our brief review of select literature (which we stress was not systematic), we did not identify any realist approaches to evaluating RI. In addition to thinking about the theoretical underpinning of evaluating research platforms, another innovation is the emerging data ecosystem that can support such evaluations. An interesting mix of suppliers—Dimensions (looking at grant data), Researchfish (tracking outcomes), Overton (identifying citations on policy documents)—complements the more traditional bibliometric suppliers (e.g. Clarivate and Scopus) in providing a lot of data that can increasingly be aligned through the DOI (Digital Object Identifier), ORCID (Open Researcher and Contributor Identifier), and GRID (Global Research Identifier Database) systems. Perhaps a next step would be to think how platforms can be both classified and identified, thus allowing them to be an explicit part of the evaluation data-ecosystem.

In suggesting the adoption of alternative paradigms to the logic model, we should stress we are not suggesting that it is “bad”, or that the others paradigms are “good” or “better”; what we are arguing is that we need to be more selective in using different paradigms based on the nature of the research that is being assessed, and suggesting that in the context of RI, the use of the realist or system approach may have advantages over the logic model that deserve being experimented with.

But overall, as research evaluators, and as funders, we should perhaps spend a bit more time and effort thinking about how we assess the impact of research platforms and in doing so move beyond our traditional comfort zones and try out new theoretical paradigms and innovate the way we capture, link, and present data.

## Conclusion

The assessment and evaluation of research is not a new field, with a number of landmark studies dating back to the 1970s and beyond [42]. It is thus perhaps a poor reflection on the field that we are dominated by a single methodological paradigm of using logic models (in various guises) to assess research impacts. This may not be too surprising given the cultural history of research funding which prioritizes the value of the project-led approach. Linear models provide the easiest way to address both the contribution challenge and the time lag challenge: simply put, it is relatively easier to link inputs to process, process to outputs, outputs to outcomes, and outcome to impact, and to measure the time lag between each of those stages. In a more practical sense, when assessing the impact of projects or programmes, this pipeline approach often works very well.

Conversely, however, the use of linear or logic models (and their derivatives) is less applicable to RIs as they provide the platform from which the projects and programmes are delivered. Moreover, research platforms are rarely given the visibility and kudos on a similar footing as research projects, despite significant investments. Given this, it is appropriate to seek or develop other evaluation paradigms to assess the impact of such platforms. As noted above, during our scan of the literature, it was notable how few studies there were using either systems approaches or realist evaluation in the context of assessing RI impact. This in part may be an artefact of historical data infrastructures—and data availability—where it is easier, say, to systematically count papers than for example viral vectors. Nevertheless, over the past 5 to 10 years, there has been somewhat of a data science revolution, meaning that in the future, as a community of people interested in assessing research, we should perhaps challenge ourselves to adopt and test different approaches using new and more innovative data sources.

The somewhat overlooked value of RI and the case for public investment has always been a topic of political debate; however, the COVID-19 pandemic has provided the most compelling evidence in support of RI. Roope and colleagues [7] articulate this, pointing to the resilience of the healthcare system and its underpinning RI (i.e. NIHR-funded BRCs, CRNs, etc.) and warns against the dangers of short-term allocation efficiency at the price of lack of capacity to meet future demands, especially if research budgets are cut to the cloth of current economic turmoil in the United Kingdom. Investments in RI are likely here to stay, and the case for taking robust and innovative approaches to quantify and qualify their impact has never been stronger.

We should stress that in writing this paper, we do not have the answers and do not know whether these alternative approaches work, but felt obliged to raise these issues for debate. In attempting to review and evaluate the impact of NIHR BRCs, especially in the context of COVID, we had a crisis of confidence in conceptualizing the BRCs within a wider biomedical and health research system and then assessing them comprehensively to derive their true value. We were left with an intellectual itch in that the current approaches to evaluating RI are not fit for purpose, and this is something that, as a community of researchers and funders, we should try to address.

## Supplementary Information

**Additional file 1.** Method for review of literature.

**Additional file 2.** Schematic diagram of the difference between a pipeline model of evaluation and the platform models of research production.

## Data Availability

The data sets used and/or analysed during the current study are available from the corresponding author on reasonable request.

## Abbreviations

BRC: Biomedical Research Centre
CBA: Cost–benefit analysis
CEA: Cost-effectiveness analysis
CMO: Context–mechanism–outcome
CRF: Clinical Research Facilities
CRN: Clinical Research Network
CTSA: Clinical and Translational Science Award
DOI: Digital Object Identifier
ESFRI: European Strategy Forum on Research Infrastructure
GRID: Global Research Identifier Database
KEF: Knowledge Exchange Framework
NIHR: National Institute for Health Research
ORCID: Open Researcher and Contributor Identifier
RECOVERY: Randomised Evaluation of COVID-19 Therapy
REF: Research Excellence Framework
RI: Research infrastructure
