# Supplementary material for: Fundamental challenges in assessing the impact of research infrastructure
Source: Health Res Policy Syst. 2021 Aug 18;19:119. doi: 10.1186/s12961-021-00769-z (PMC8371591; doi:10.1186/s12961-021-00769-z)
Supplement: Supplementary file 1 — Additional file 1. Method for review of literature. [file 12961_2021_769_MOESM1_ESM.docx]

**Method for review of literature**

Search terms used in google scholar and grey literature were ‘research infrastructure’ ‘research infrastructure impact and/or assessment’, ‘methods for evaluating research infrastructure’, ‘research infrastructure evaluation’ and ‘evaluation (of) research infrastructure’. Search parameters were set to the top 50 items between 2012-2018.

Initially, searching for ‘research infrastructure’ yielded 74000 items. The search was then narrowed down resulting in 34 articles/reports for ‘research infrastructure impact and or/assessment’, 11 for ‘research infrastructure evaluation’ and 17 results ‘evaluation (of) research infrastructure’. No results were found for ‘methods of evaluating research infrastructure’. Additional searches of grey literature produced 15 reports and articles in total.

After deduplication and an initial review, 34 publications were included in the analysis based on content. A content analysis approach was taken to synthesise methods of preference for impact assessment of research infrastructure. The literature analysed included meta-analyses and scoping reviews which detailed previous methodologies. This allowed the inclusion of a wide breadth of methods within a narrow search parameter of the literature review. The literature was reordered and assessed in a chronological fashion to assess the evolution of methodologies. It is to be noted that this isn’t a systematic literature review and may represent a biased selection based on publication categorisation and search engine analytics.
