# Supplementary material for: Fundamental challenges in assessing the impact of research infrastructure
Source: Health Res Policy Syst. 2021 Aug 18;19:119. doi: 10.1186/s12961-021-00769-z (PMC8371591; doi:10.1186/s12961-021-00769-z)
Supplement: Supplementary file 2 — Additional file 2. Schematic diagram of the difference between a pipeline model of evaluation and the platform models of research production. [file 12961_2021_769_MOESM2_ESM.pptx]

## Slide 1
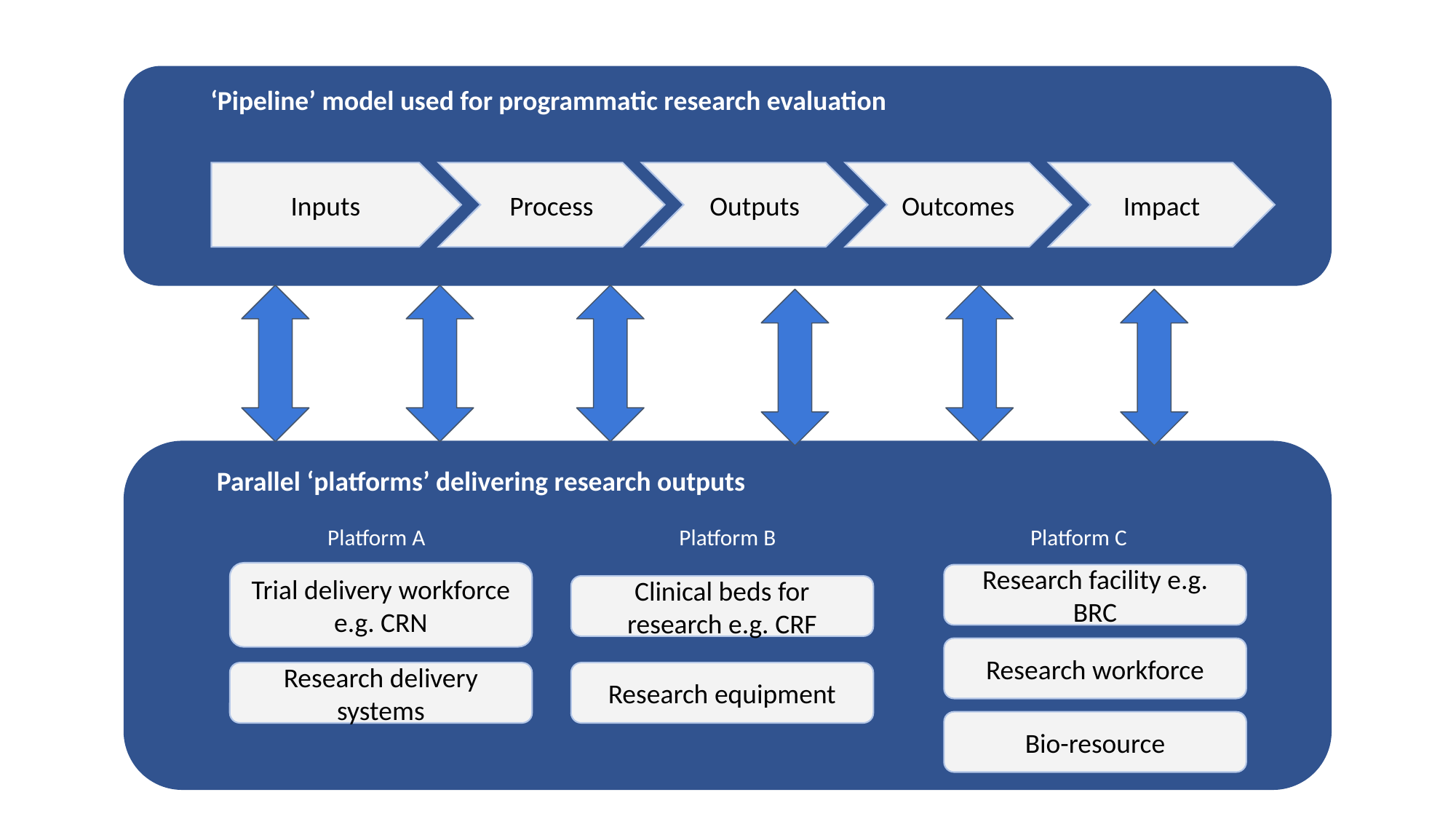

‘Pipeline’ model used for programmatic research evaluation
Outcomes
Impact
Outputs
Inputs
Process
Parallel ‘platforms’ delivering research outputs
Platform A
Platform B
Platform C
Trial delivery workforce e.g. CRN
Research facility e.g. BRC
Clinical beds for research e.g. CRF
Research workforce
Research delivery systems
Research equipment
Bio-resource
